# Diagnostic accuracy of *Mycobacterium tuberculosis* cell-free DNA for tuberculosis: A systematic review and meta-analysis

**DOI:** 10.1371/journal.pone.0253658

**Published:** 2021-06-23

**Authors:** Guocan Yu, Yanqin Shen, Bo Ye, Yan Shi

**Affiliations:** Zhejiang Tuberculosis Diagnosis and Treatment Center, Affiliated Hangzhou Chest Hospital, Zhejiang University School of Medicine, Hangzhou, Zhejiang, China; School of Medicine, Tehran University of Medical Sciences, ISLAMIC REPUBLIC OF IRAN

## Abstract

**Background:**

Diagnosis of tuberculosis (TB) is still difficult. The purpose of our study was to evaluate the diagnostic accuracy of *Mycobacterium tuberculosis* cell-free DNA (cfDNA) for diagnosing of TB.

**Methods:**

We searched relevant databases for studies that used cfDNA to diagnose TB. We evaluated the accuracy of cfDNA compared with the composite reference standard (CRS) and culture. True positive, false positive, false negative, and true negative values for cfDNA were obtained first, then the estimated pooled sensitivity, specificity, positive predictive value (PPV), negative predictive value (NPV), diagnostic odds ratio (DOR), and the area under the summary receiver operating characteristic (SROC) curve (AUC) of cfDNA for diagnosing TB were calculated with 95% confidence intervals (CIs). Heterogeneity was determined using the I^2^ statistic. When the heterogeneity was obvious, the source of heterogeneity was further discussed.

**Results:**

We included 14 independent studies comparing cfDNA with the CRS, and 4 studies compared with culture. The pooled sensitivity, specificity, PPV, NPV, DOR, and AUC of the SROC were 68%, 98%,99%, 62%, 83, and 0.97 as compared with the CRS, respectively. The pooled sensitivity, specificity, PPV, NPV, DOR, and AUC of the SROC were 48%, 91%, 92%, 60%, 5, and 0.88 as compared with culture, respectively. The heterogeneity between studies was significant.

**Conclusions:**

The accuracy of cfDNA testing for TB diagnosis was good compared with CRS and culture. cfDNA can be used for rapid early diagnosis of TB.

## Introduction

Tuberculosis [TB] is a chronic infectious disease caused by *Mycobacterium tuberculosis* (MTB) that can involve any organ or system in the body [1]. Ten million new cases of TB and approximately 1.5 million TB-related deaths occurred in 2019, illustrating the global public health importance of the disease [2]. MTB most commonly infects the lungs, causing pulmonary tuberculosis (PTB); infection of organs other than the lungs is known as extrapulmonary tuberculosis (EPTB) [3, 4]. Rapid early diagnosis of PTB and EPTB is critical to control the disease and prevent its transmission, as delayed diagnosis leads to delayed treatment and enables disease spread [5]. However, rapid early diagnosis is difficult because of the low sensitivity of the acid-fast bacillus (AFB) smear, long MTB culture time, and atypical diagnostic imaging findings [6]. Although modern immunology and molecular biology techniques have improved diagnostic sensitivity and detection speed compared to classical microbiological methods, many problems remain regarding standardization of testing results and applicability of testing in various populations [7]. Breaking the TB transmission cycle requires the development of a rapid, accurate, and highly effective diagnostic test.

Cell-free DNA (cfDNA) is composed of extracellular DNA fragments released from original cells and exists in a cell-free state in various body fluids [8]. First discovered in human plasma by Mandel and Metais in 1948, cfDNA can be detected in human plasma, synovial fluid, cerebrospinal fluid (CSF), pleural fluid, urine, prostate fluid, saliva, and other body fluids [9–11]. Use of its detection has enabled great strides in prenatal diagnosis and the diagnosis and therapeutic monitoring of cancer and other diseases [12, 13]. Recent studies have also found that cfDNA is present in a variety of pathogenic bacterial, fungal, and parasitic infections, showing its value in the diagnosis and treatment of infectious diseases [11, 14]. MTB cfDNA can be detected with high diagnostic performance within a few hours in human plasma, pleural fluid, and other body fluids by polymerase chain reaction (PCR), enabling a new approach to TB diagnosis [10, 15]. However, the diagnostic efficacy of cfDNA applications in TB is remains controversial. Therefore, we performed this systematic review and meta-analysis to assess the accuracy and utility of cfDNA testing for the early diagnosis of TB.

## Methods

### Design and registration

This meta-analysis followed the Preferred Reporting Items for Systematic Reviews and Meta-Analysis for Diagnostic Test Accuracy (PRISMA-DTA) guidelines [16] and was registered in the International Platform of Registered Systematic Review and Meta-Analysis Protocols (registration number 2020110101) [17].

### Information sources

We searched the Pubmed, Cochrane Library, Embase, China National Knowledge Infrastructure, and Wanfang databases for studies that used cfDNA to diagnose TB on November 19, 2020. References cited in the identified studies were also evaluated to identify additional studies.

### Search strategy

Two investigators (GCY and YQS) designed and implemented the comprehensive search strategy for studies published in the English or Chinese language. Search strategy of PubMed was listed as follows:

#1 "Tuberculosis"[Mesh] OR “Tuberculoses Kochs Disease” OR “Koch’s Disease” OR “Koch Disease” OR “Mycobacterium tuberculosis Infection” OR “Infection, Mycobacterium tuberculosis” OR “Infections, Mycobacterium tuberculosis” OR “Mycobacterium tuberculosis Infections”
#2 "Cell-Free Nucleic Acids"[Mesh] OR “Cell Free Nucleic Acids” OR “Nucleic Acids, Cell-Free” OR “Circulating Cell-Free Nucleic Acids” OR “Circulating Cell Free Nucleic Acids” OR “Circulating Nucleic Acids” OR “Acids, Circulating Nucleic” OR “Nucleic Acids, Circulating” OR “Cell-Free Nucleic Acid” OR “Cell Free Nucleic Acid” OR “Nucleic Acid, Cell-Free” OR “Cell-Free DNA” OR “Cell Free DNA” OR “DNA, Cell-Free” OR cfDNA OR cirDNA OR “Cell-Free Deoxyribonucleic Acid” OR “Acid, Cell-Free Deoxyribonucleic” OR “Cell Free Deoxyribonucleic Acid” OR “Deoxyribonucleic Acid, Cell-Free” OR “Circulating DNA” OR “DNA, Circulating” OR “Cell-Free RNA” OR “Cell Free RNA” OR “RNA, Cell-Free” OR cfRNA OR cirRNA OR “Cell-Free Ribonucleic Acid” OR “Acid, Cell-Free Ribonucleic” OR “Cell Free Ribonucleic Acid” OR “Ribonucleic Acid, Cell-Free” OR “Circulating RNA” OR “RNA, Circulating”
#3 #1 AND #2

The other four databases used a similar search strategy.

### Eligibility criteria

#### Types of studies

Any type of study design, such as retrospective, prospective, or case-control, were eligible for inclusion provided the study assessed the efficacy of cfDNA to diagnose TB. Studies that reported only sensitivity or specificity were excluded.

#### Participants

Participants of any ethnicity, sex, or age were included provided cfDNA was used to diagnose and confirm TB infection.

#### Index tests

cfDNA was considered the index test.

#### Outcomes

The sensitivity and specificity of the index test were considered the primary outcome. Sensitivity is the probability that the index test will be positive in an infected patient. Specificity is the probability that the index test will be negative in a noninfected patient [18].

#### Comparator test

Single-arm studies were also included if their participants, index test, and outcomes met the inclusion criteria. Comparator tests were not mandatory.

#### Target conditions

Studies that diagnosed TB using cfDNA with clear reference standards and had their full text accessible were included. True positive (TP), false positive (FP), false negative (FN), and true negative (TN) values for the index test were extracted directly or calculated from the original studies. Studies reported in languages other than English or Chinese, those with <10 participants, conference abstracts without full articles, and case reports were excluded.

#### Reference standards

MTB culture or a composite reference standard (CRS) was defined as the reference standard. The CRS included MTB AFB smear, culture, clinical manifestations, radiographic changes, other nucleic acid amplification tests, immunological tests, and response to anti-TB treatment.

### Literature screening and selection

The candidate studies were imported into ENDNOTE X9.2 literature management software. The same two investigators (GCY and YQS) independently evaluated the imported studies for selecting eligible articles based on inclusion criteria by reviewing their titles and abstracts and then the full text. If there was disagreement between the two investigators, they consulted with a third investigator (YS). First, we gave the controversial literature to a third researcher for independent evaluation, then the three investigators discussed and reported the reasons for inclusion or exclusion, respectively, and then the literature was included or excluded according to the inclusion and exclusion criteria after obtaining agreement.

### Data extraction

We extracted the following information from each study: first author name; publication date; country; TP, FP, FN, and TN values for the index test; type of study design; patient selection method; specimen types; target gene; PCR method; specimen condition; TB types; and other parameters. Data was independently extracted by the same two investigators who screened the literature. Any disagreement was resolved by consultation with the third investigator.

### Quality evaluation

Two investigators independently evaluated study quality using the revised Quality Assessment of Diagnostic Accuracy Studies tool (QUADAS-2) [19]. Any disagreement was handled as noted above. Publication bias was not assessed, as per the PRISMA-DTA guidelines [16]. The Grading of Recommendations Assessment, Development and Evaluation (GRADE) guideline was used to assess the strength of the body of evidence [20].

### Data synthesis and statistical analysis

When different types of TB, target genes or PCR methods were reported in the same article, we considered them as separate studies. TP, FP, FN, and TN values for the index test in each included study were obtained first, then the estimated pooled sensitivity and specificity of cfDNA for diagnosing TB were calculated with 95% confidence intervals (CIs). Calculations were performed using Stata software version 15.0 (Stata Corp., College Station, TX, USA) with the *midas* command package or Meta-DiSc software version 1.4 (XI Cochrane Colloquium, Barcelona, Spain). Forest plots for sensitivity and specificity were generated using Review Manager (RevMan) software version 5.3 (Nordic Cochrane Centre, Copenhagen, Denmark). We also calculated the pooled positive predictive value (PPV), negative predictive value (NPV), diagnostic odds ratio (DOR), and the area under the summary receiver operating characteristic (SROC) curve (AUC). Heterogeneity was determined using the *I*^*2*^ statistic (0% indicated no observed heterogeneity; <50% indicated minor heterogeneity; >50% indicated substantial heterogeneity) [21]. Subgroup, meta-regression, and sensitivity analyses were used to explore sources of heterogeneity when significant heterogeneity was observed. Subgroup and meta-regression analyses were performed according to TB type, specimen type, target gene, PCR method, type of study design, specimen condition, and patient selection method. At least 4 eligible studies were required to perform the meta-analysis and meta-regression analyses for predefined variable parameters; these analyses were performed using Stata software version 15.0 with the *midas* command package [22].

## Results

### Identification of studies and study characteristics

**Fig 1:**
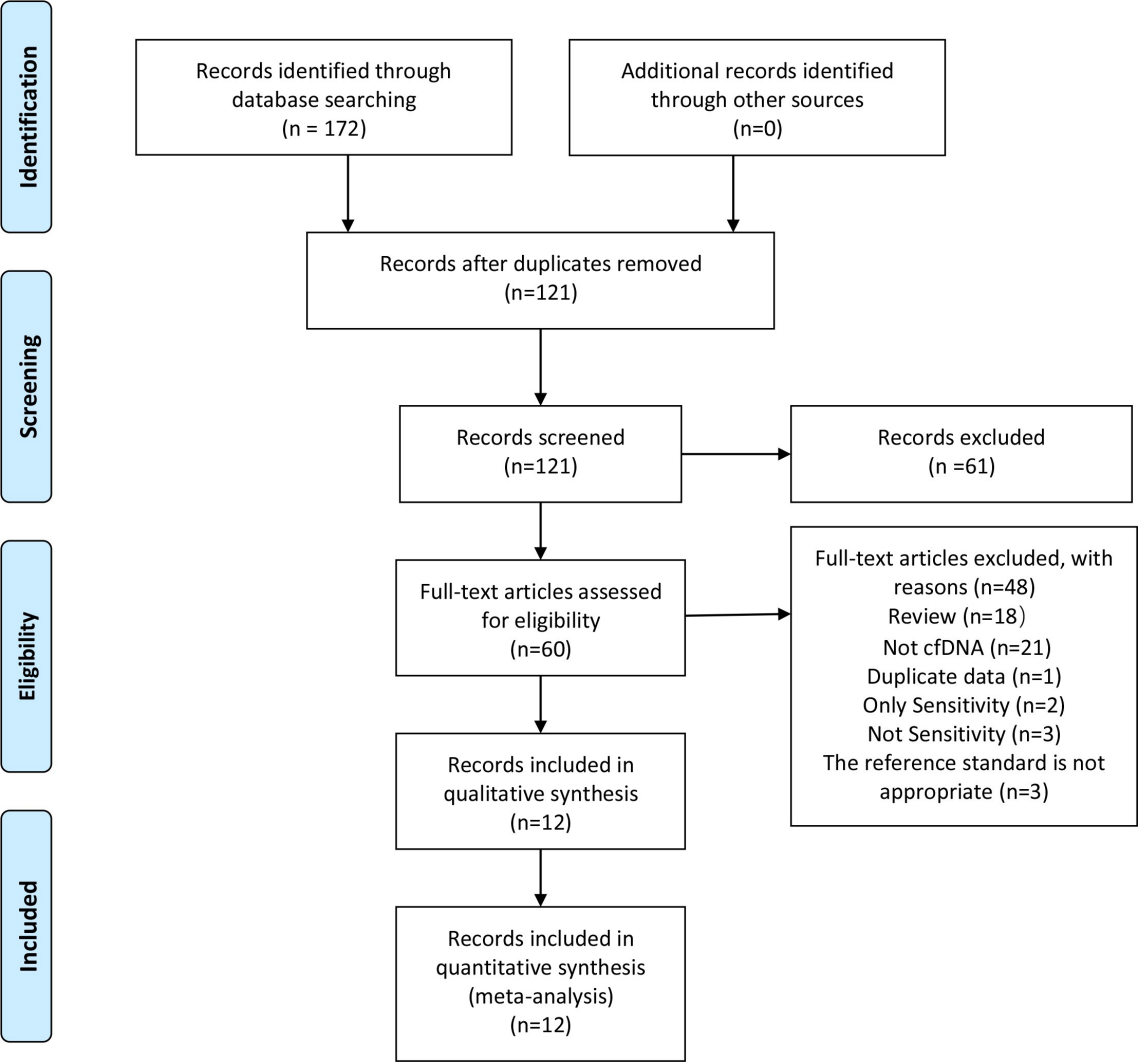
Flow chart of literature retrieval. cfDNA, cell free DNA.

Using our designed search strategy, 172 candidate articles were obtained from the databases. We identified twelve eligible articles according to the inclusion criteria by screening the title, abstract, and full text (Fig 1) [5, 9, 15, 23–31]. The kappa index of agreement between the two investigators for selection and data extraction was 0.785 (95% CI, 0.581–0.989). Two articles that reported sensitivity without specificity were excluded [32, 33]. We also excluded three articles that did not report sensitivity and specificity [34–36] and one article that reported data from another included article [37]. The reference criteria used in three articles did not meet the inclusion criteria of this study and were excluded [10, 38, 39]. One article that simultaneously reported the efficacy of different PCR methods to detect cfDNA for PTB and EPTB diagnosis was considered as four separate studies [5], and another article that simultaneously reported the efficacy of different target gene to detect cfDNA for PTB diagnosis was considered as two separate studies [15]. Another simultaneously reported the efficacy of cfDNA for PTB and EPTB diagnosis [28]. Therefore, 18 studies were included for analysis (14 studies used CRS as the reference standard and 4 studies used MTB culture as the reference standard). Included studies were analyzed separately according to different reference standards. The included studies and their characteristics were presented in Table 1. 15 studies were conducted in TB endemic areas, 13 in China, 1 in India, and 1 in South Africa. Two studies were reported in Chinese [23, 28] and the remaining ones were reported in English. All studies focused on adults. Only one study included a subset of HIV-positive patients [31]. Ten were case-control studies, seven were prospective, and one was cohort study. The specimens used in the articles were blood, urine, CSF, pleural fluid, and peritoneal fluid. The number of samples ranged from 19 to 412 and the average sample size was 124.3.

**Table 1:** Characteristics of the included studies.

Author	Year	County	Sample type	Reference	Diagnosis	Target gene	PCR method	N	TP	FP	FN	TN	Type of research	Sample condition	Patient selection method
Che, N.	2017	China	Pleural effusion	CRS	Tuberculosis pleurisy	IS6110	Real-Time PCR	78	45	0	15	18	prospective	Frozen	Consecutive
Yang, J.a	2017	China	Plasma	CRS	Pulmonary TB	IS6110	Digital PCR	56	28	0	0	28	Case-control	Frozen	Convenience
Yang, J.b	2017	China	Plasma	CRS	Extrapulmonary TB	IS6110	Digital PCR	56	28	0	0	28	Case-control	Frozen	Convenience
Yang, J.c	2017	China	Plasma	CRS	Pulmonary TB	IS6110	Real-Time PCR	56	14	0	14	28	Case-control	Frozen	Convenience
Yang, J.d	2017	China	Plasma	CRS	Extrapulmonary TB	IS6110	Real-Time PCR	56	15	0	13	28	Case-control	Frozen	Convenience
Shou, J.	2018	China	Pleural effusion	CRS	Tuberculosis pleurisy	Unreported	Real-Time PCR	136	65	0	38	33	prospective	Fresh	Consecutive
Li, X.	2020	China	Cerebrospinal fluid	CRS	Tuberculosis meningitis	IS6110	Real-Time PCR	68	26	0	20	22	prospective	Fresh	Consecutive
Lyu, L.a	2020	China	Plasma	CRS	Pulmonary and extrapulmonary TB	IS6110	Digital PCR	261	63	7	92	99	Case-control	Frozen	Consecutive
Lyu, L.b	2020	China	Plasma	CRS	Pulmonary and extrapulmonary TB	IS1081	Digital PCR	261	42	7	113	99	Case-control	Frozen	Consecutive
Shao, L.	2020	China	Cerebrospinal fluid	CRS	Tuberculosis meningitis	IS6110	Real-Time PCR	84	32	0	28	24	prospective	Frozen	Consecutive
Sharma, P.	2020	India	Ascitic fluid	CRS	Abdominal tuberculosis	devR	Real-Time PCR	65	22	1	33	9	prospective	Frozen	Consecutive
Yang, X.	2020	China	Pleural effusion	CRS	Tuberculosis pleurisy	IS6110	Real-Time PCR	286	153	0	68	65	prospective	Frozen	Consecutive
Han, B.a	2020	China	Plasma	CRS	Pulmonary TB	Unreported	Real-Time PCR	140	63	3	16	58	Case-control	Frozen	Convenience
Han, B.b	2020	China	Plasma	CRS	Extrapulmonary TB	Unreported	Real-Time PCR	100	31	3	8	58	Case-control	Frozen	Convenience
Ushio, R.a	2016	Japan	Plasma	Culture	Pulmonary TB	IS6110	Digital PCR	52	21	1	12	18	Case-control	Frozen	Convenience
Ushio, R.b	2016	Japan	Plasma	Culture	Pulmonary TB	gyrB	Digital PCR	52	10	0	23	19	Case-control	Frozen	Convenience
Labugger, I.	2017	Germany	Urine	Culture	Pulmonary TB	IS6110	Real-Time PCR	19	7	0	4	8	Prospective	Frozen	Consecutive
Patel, K.	2018	South Africa	Urine	Culture	Pulmonary TB	DR region	Real-Time PCR	412	75	27	100	210	Cohort study	Frozen	Convenience

PCR, polymerase chain reaction; TP, true positive; FP, false positive; FN, false negative; TN, true negative; CRS, composite reference standard; TB, tuberculosis; DR, direct repeat.

### Study quality

**Fig 2:**
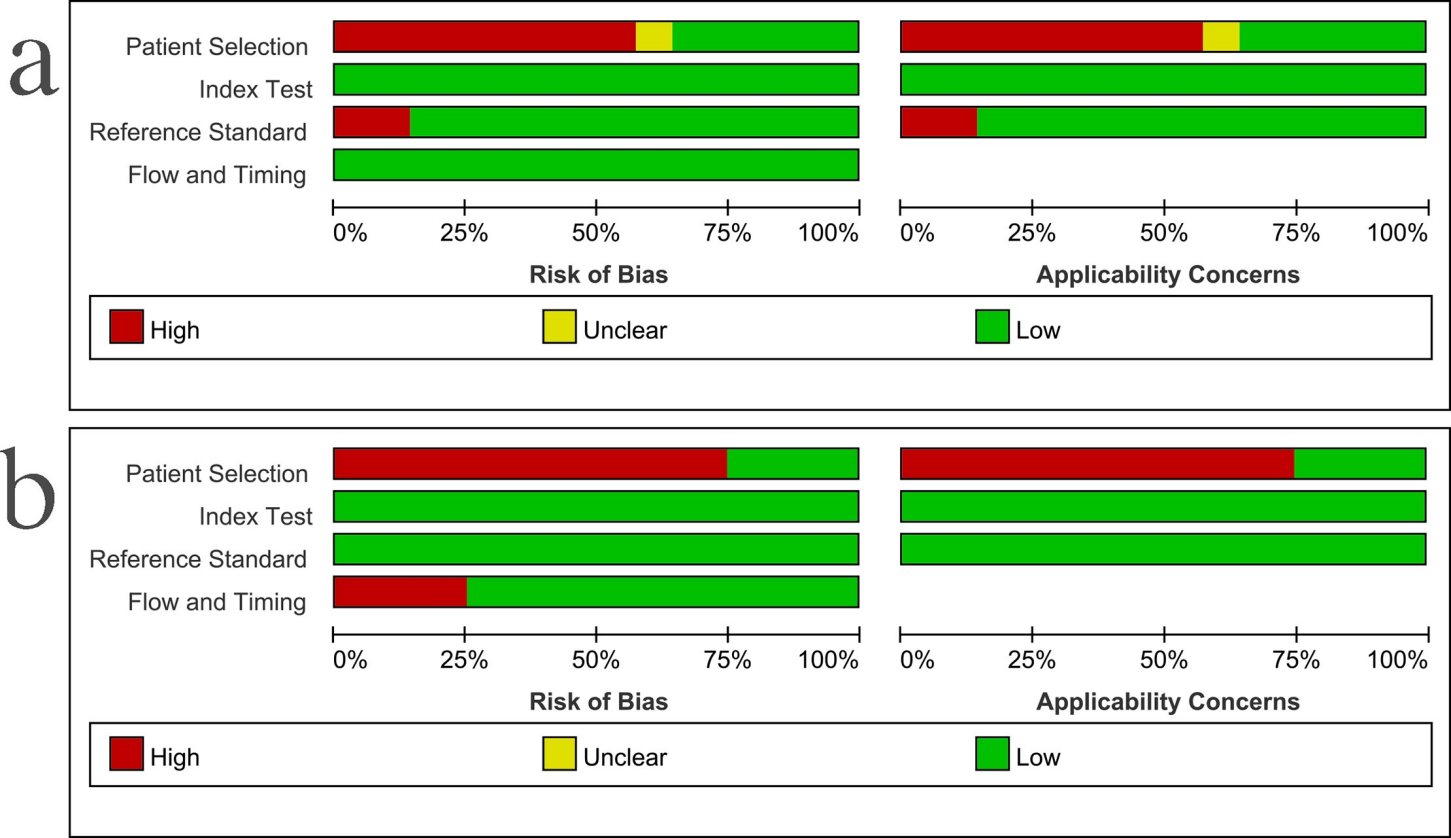
Methodological quality graphs (risk of bias and applicability concerns), a) compared with the composite reference standard, b) compared with culture.

The results of the methodological study quality assessment compared with CRS and culture were shown in Fig 2. When using CRS as the reference standard, six studies had nonconsecutive patient selection, one had unclear inappropriate exclusions, and four did not include treatment response in its reference criteria; these were the major sources of bias. The risk of bias from the index test and the flow and timing was judged to be relatively low. When using culture as the reference standard, three studies had nonconsecutive patient selection, one had high bias from the flow and timing; these were the major sources of bias. The risk of bias from the index test and the reference standard was judged to be relatively low. According to the GRADE guidelines, the quality of evidence of this meta-analysis was moderate.

### Diagnostic accuracy of cfDNA for TB

**Fig 3:**
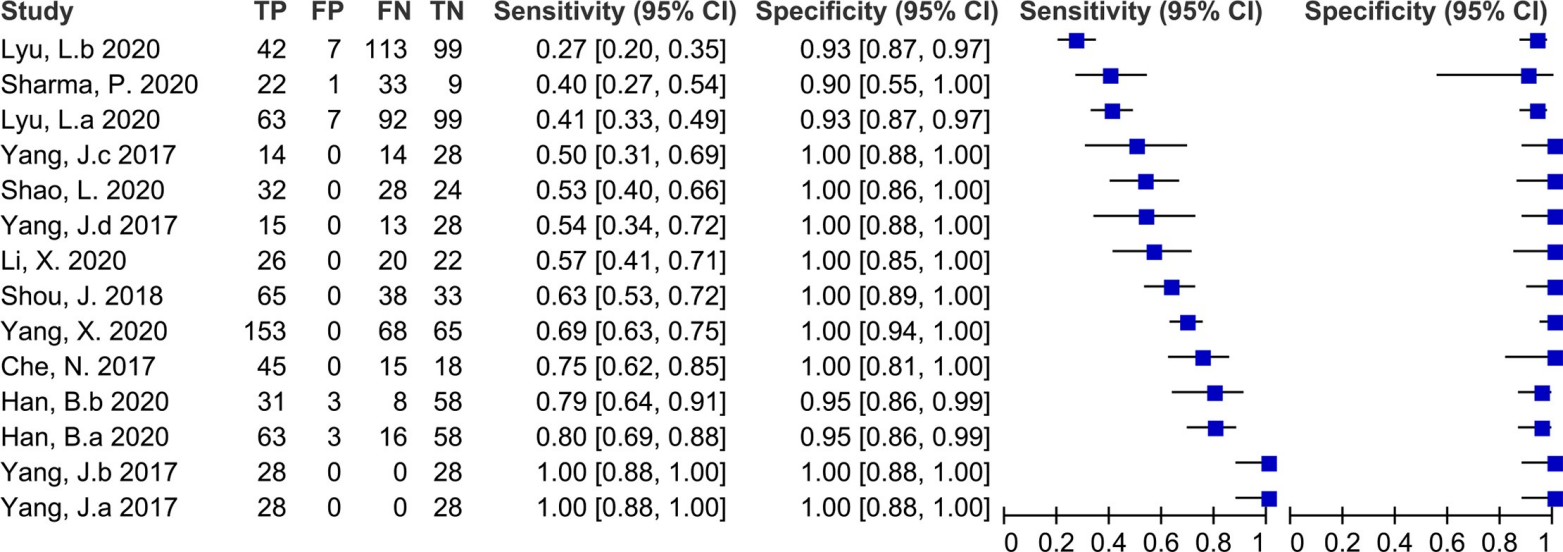
Forest plot for the sensitivity and specificity of cell free DNA for the diagnosis of tuberculosis compared with a composite reference standard.

Fourteen studies with 1703 participants evaluated the accuracy of cfDNA for TB diagnosis compared with CRS. The sensitivity of cfDNA ranged from 27% (20%–35%) to 100% (88%–100%). The pooled sensitivity was 68% (52%–80%) and the *I*^2^ value was 94%. The specificity of cfDNA ranged from 90% (55%–100%) to 100% (94%–100%). The pooled specificity was 98% (95%–99%) and the *I*^2^ value was 72% (Fig 3). The heterogeneity of sensitivity and specificity was significant. The pooled PPV, NPV, DOR, and AUC of the SROC were 99% (99–100%), 62% (50–75%), 83 (25–278), and 0.97 (0.95–0.98), respectively.

**Fig 4:**
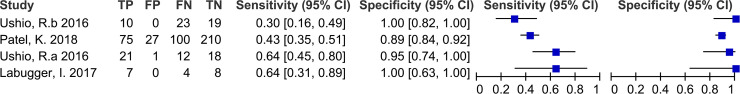
Forest plot for the sensitivity and specificity of cell free DNA for the diagnosis of tuberculosis compared with culture.

Four studies with 535 participants evaluated the accuracy of cfDNA for TB diagnosis compared with culture. All four studies included PTB and did not include EPTB, two studies used plasma specimens and the other two studies used urine specimens. The sensitivity ranged from 30% (16%–49%) to 64% (31%–89%). The specificity ranged from 89% (84%–192%) to 100% (82%–100%) (Fig 4). The pooled sensitivity, specificity, PPV, NPV, DOR, and AUC of the SROC were 48% (35–62%), 91% (81–96%), 92% (80–100%), 60% (49–72%), 5 (3–7), and 0.88 (0.85–0.90), respectively. The heterogeneity between studies was significant.

### Meta-regression and subgroup analyses

Studies that did not report target genes were excluded from the subgroup and meta-regression analysis. When compared with CRS, the results of the meta-regression analysis are presented in Table 2. TB type (PTB or EPTB), specimen type (plasma or non-plasma), PCR method (real-time PCR or digital PCR), and type of study design (prospective or case-control) had no effect on sensitivity and specificity of cfDNA testing when compared with CRS (*P* >0.05). Although target gene (IS6110 or non- IS6110 [IS1081, devR]) and specimen condition (fresh or frozen) had no effect on sensitivity (*P* >0.05), they did significantly affect specificity (*P* <0.05). Patient selection method (convenience or consecutive) significantly affected sensitivity (*P* <0.05), but had no effect on specificity (*P* >0.05). However, when compared with culture, the number of included studies was limited and meta-regression analysis could not be performed.

**Table 2:** Meta-regression analysis for different parameters compared with composite reference standard.

Variables	Subgroup	No. of studies	No. of specimens	Sensitivity (95% CI)	Meta regression p value	Specificity (95% CI)	Meta regression p value
TB type	PTB	3	252	83% (64–100%)	0.56	99% (96–100%)	0.05
	EPTB	9	929	68% (53–84%)		99% (98–100%)	
Specimen type	Plasma	8	986	73% (56–91%)	0.28	97% (94–99%)	0.05
	Non-Plasma	6	717	60% (37–83%)		99% (98–100%)	
Target gene	IS6110	9	1001	72% (56–88%)	0.07	100% (99–100%)	<0.01
	Non-IS6110	2	326	32% (2–66%)		96% (78–100%)	
PCR method	Real-Time PCR	10	1069	63% (45–81%)	0.28	98% (96–100%)	0.36
	Digital PCR	4	634	80% (56–100%)		97% (93–100%)	
Study design	Prospective	6	717	60% (37–83%)	0.28	99% (98–100%)	0.05
	Case-control	8	986	73% (56–91%)		97% (94–99%)	
Specimen condition	Fresh	2	204	60% (20–100%)	0.66	100% (99–100%)	0.01
	Frozen	12	1499	69% (53–84%)		97% (95–99%)	
Patient selection method	Convenience	6	464	83% (71–95%)	0.03	98% (96–100%)	0.14
	Consecutive	8	1239	53% (37–69%)		97% (95–100%)	

CI, confidence interval; TB, tuberculosis; PTB, pulmonary tuberculosis; EPTB, extrapulmonary tuberculosis; PCR, polymerase chain reaction.

**Fig 5:**
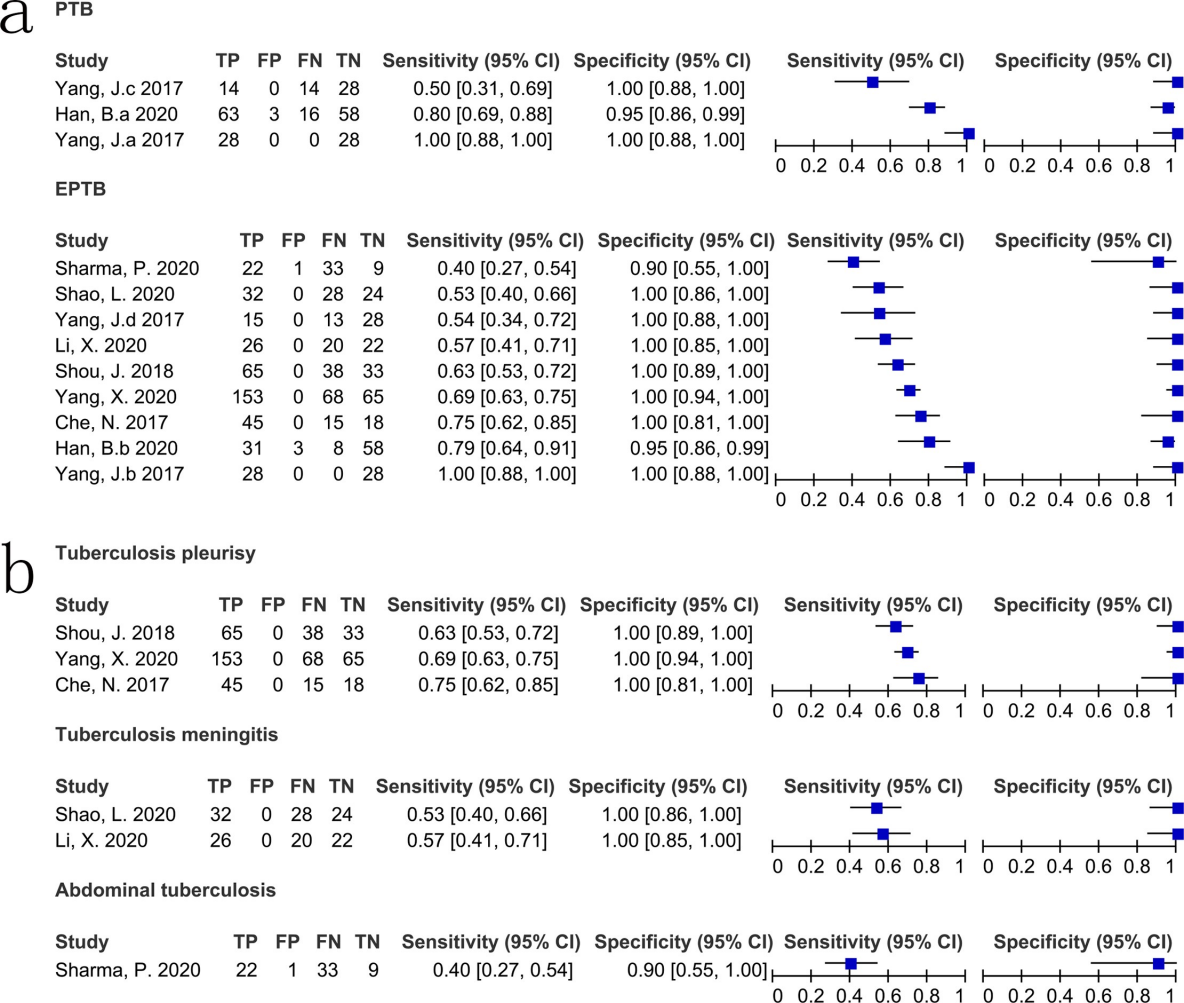
Forest plot for the sensitivity and specificity of cell free DNA for the diagnosis of different types of tuberculosis compared with a composite reference standard. a) pulmonary tuberculosis and extrapulmonary tuberculosis. b) different types of extrapulmonary tuberculosis. PTB, pulmonary tuberculosis; EPTB, extrapulmonary tuberculosis.

When compared with CRS, the case-control studies all used plasma specimens for testing, so the results of the subgroup analyses were the same for these two parameters. The sensitivity, specificity, PPV, NPV, DOR, and AUC of cfDNA testing using plasma specimens in case-control studies were 78% (44%–94%; *I*^2^ = 98%), 97% (93%–99%; *I*^2^ = 88%), 97% (95–100%; *I*^2^ = 61.5%), 75% (62–88%; *I*^2^ = 98.3%), 12 (8–18; *I*^2^ = 51.2%), and 0.98 (0.96–0.99), respectively. The sensitivity, specificity, PPV, NPV, DOR, and AUC of cfDNA testing using non-plasma specimens in prospective studies were 60% (51%–69%; *I*^2^ = 79%), 100% (57%–100%; *I*^2^ = 67%), 100% (100–100%; *I*^2^ = 0.0%), 45% (35–54%; *I*^2^ = 70.3%), 97 (15–615; *I*^2^ = 63.7%), and 0.82 (0.78–0.85), respectively. The heterogeneity between studies was still significant. The sensitivity, specificity, PPV, NPV, DOR, and AUC of cfDNA testing using frozen specimens were 70% (50%–84%; *I*^2^ = 96%), 97% (94%–99%; *I*^2^ = 76%), 99% (98–100%; *I*^2^ = 52.1%), 65% (51–78%; *I*^2^ = 98.4%), 16 (10–24; *I*^2^ = 54.5%), and 0.97 (0.96–0.98), respectively. The sensitivity, specificity, PPV, NPV, DOR, and AUC of cfDNA testing using IS6110 as the target gene were 74% (51%–88%; *I*^2^ = 95%), 100% (95%–100%; *I*^2^ = 90%), 100% (99–100%; *I*^2^ = 0.0%), 66% (53–79%; *I*^2^ = 98.1%), 31 (15–68; *I*^2^ = 59.2%), and 1.00 (0.99–1.00), respectively. Heterogeneity in sensitivity and specificity was still significant. Detection of cfDNA using real-time PCR exhibited pooled sensitivity, specificity, PPV, NPV, DOR, and AUC of 64% (55%–71%; *I*^2^ = 78%), 99% (96%–100%; *I*^2^ = 27%), 100% (100–100%; *I*^2^ = 0.0%), 57% (44–71%; *I*^2^ = 92.4%), 38 (17–87; *I*^2^ = 16.9%), and 0.95 (0.93–0.97), respectively. Detection of cfDNA using digital PCR exhibited pooled sensitivity, specificity, PPV, NPV, DOR, and AUC of 44% (39%–49%; *I*^2^ = 95%), 99% (91%–97%; *I*^2^ = 56%), 96% (92–100%; *I*^2^ = 78.6%), 75% (56–94%; *I*^2^ = 99.2%), 9 (5–14; *I*^2^ = 62.2%), and 1.00 (0.90–1.00), respectively. For PTB, the sensitivity of cfDNA testing ranged from 50% (31%–69%) to 100% (88%–100%) and specificity ranged from 95% (86%–99%) to 100% (88%–100%). For EPTB, the sensitivity ranged from 40% (27%–54%) to 100% (88%–100%) and the specificity ranged from 90% (55%–100%) to 100% (94%–100%) (Fig 5A). The diagnostic accuracy of cfDNA testing according to TB type (PTB and EPTB) was presented in Table 3. Table 3 and Fig 5B showed the accuracy according to EPTB type (pleurisy, meningitis, and abdominal).

**Table 3:** Subgroup analysis for different types of tuberculosis compared with a composite reference standard.

TB type	Specimen type	No. of studies	No. of specimens	Sensitivity (95% CI)	Specificity (95% CI)	PPV (95% CI)	NPV (95% CI)	DOR (95% CI)	AUC (95% CI)
Pulmonary TB	Plasma	3	252	78% (70–84%)	97% (93–99%)	99% (97–100%)	82% (62–100%)	29 (10–85)	0.99 (0.97–1.00)
Extrapulmonary TB	All	9	929	65% (61–69%)	99% (96–100%)	100% (100–100%)	59% (38–80%)	61 (20–185)	0.98 (0.93–1.00)
	Plasma	3	212	78% (68–86%)	97% (93–99%)	99% (96–100%)	87% (72–100%)	35 (10–115)	0.99 (0.97–1.00)
	Non-plasma	6	717	63% (59–67%)	99% (97–100%)	100% (100–100%)	45% (35–54%)	97 (15–615)	0.82 (0.78–0.85)
	Pleural effusion (Tuberculosis pleurisy)	3	500	68% (64–73%)	100% (97–100%)	100% (100–100%)	49% (43–55%)	54 (11–268)	0.90 (0.35–1.00)
	Cerebrospinal fluid (Tuberculosis meningitis)	2	152	55% (45–64%)	100% (92–100%)	100% (98–100%)	49% (39–59%)	26 (4–184)	0.77 (0.70–0.84)
	Ascitic fluid (Abdominal tuberculosis)	1	65	40% (27–54%)	90% (56–100%)	96% (87–100%)	21% (9–34%)	4 (1–26)	0.65 (0.52–0.76)

TB, tuberculosis; PPV, positive predictive value; NPV, negative predictive value; DOR, diagnostic odds ratio, AUC, the area under the summary receiver operating characteristic curve.

When compared with culture, the sensitivity, specificity, PPV, NPV, DOR, and AUC of cfDNA testing using plasma specimens for digital PCR were 47% (35–60%; *I*^2^ = 86.7%), 97% (86–100%; *I*^2^ = 29.2%), 98% (93–100%; *I*^2^ = 0.0%), 52% (38–66%; *I*^2^ = 36.2%), 18 (3–124; *I*^2^ = 0.0%), and 0.72 (0.63–0.81), respectively. The sensitivity, specificity, PPV, NPV, DOR, and AUC of cfDNA testing using urine specimens for real-time PCR were 44% (37–52%; *I*^2^ = 44.6%), 89% (84–93%; *I*^2^ = 47.4%), 86% (61–100%; *I*^2^ = 93.8%), 68% (63–73%; *I*^2^ = 0.0%), 4 (3–6; *I*^2^ = 0.0%), and 0.67 (0.62–0.71), respectively.

Sensitivity analysis did not identify specific articles as sources of heterogeneity in sensitivity and specificity.

## Discussion

This meta-analysis evaluated the effectiveness of using cfDNA testing to diagnose TB. Rapid early diagnosis is essential to improve TB prognosis and control transmission. cfDNA testing had an overall sensitivity of 68%, specificity of 98%, DOR of 83, and AUC of 0.97 for TB diagnosis (including PTB and EPTB) compared with CRS, and sensitivity of 48%, specificity of 91%, DOR of 5, and AUC of 0.88 compared with culture, demonstrating excellent diagnostic performance. There was significant heterogeneity in sensitivity and specificity. The acquisition of the diagnosis in different types of TB is distinctive considering the differences in characteristics between PTB and EPTB, and even between different types of EPTB. Therefore, we analyzed the different types of TB separately.

When compared with CRS, the sensitivity, specificity, DOR, and AUC of cfDNA testing using plasma for diagnosis of PTB were 78%, 97%, 29, and 0.99, respectively; for diagnosis of EPTB, the sensitivity, specificity, DOR, and AUC of cfDNA testing were 65%, 99%, 61, and 0.98, respectively. These results showed that cfDNA testing has good diagnostic performance for both PTB and EPTB. For PTB, the sensitivity of cfDNA testing was higher compared with EPTB, while the specificity was lower; however, the overall diagnostic efficacy was similar. Respiratory specimens, such as sputum and bronchoalveolar lavage fluid (BALF), are typically used to diagnose PTB but obtaining these specimens can be difficult, particularly in children [40]. Moreover, the diagnosis of PTB is difficult when the patient does not cough up sputum or is unable to collect sputum of acceptable quality. Although it may be possible to obtain BALF via fiberoptic bronchoscopy, this invasive procedure may be poorly tolerated by patients with milder disease. The use of non-respiratory specimens such as gastric or stool specimens to diagnose PTB has also been reported but remains controversial [41, 42]. Plasma-based cfDNA assay testing offers a new pathway for PTB diagnosis. Although plasma-based testing such as the interferon gamma release assay may provide indirect evidence of PTB without showing evidence of MTB [43], plasma-based cfDNA testing can provide direct evidence of MTB. Plasma specimens are easily accessible and widely applicable. For PTB patients in whom respiratory specimens are difficult to obtain, plasma cfDNA testing is a convenient and effective alternative diagnostic method. However, the diagnostic efficacy of cfDNA testing using plasma for the diagnosis of PTB was reduced when culture was the reference standard, which may be related to the inconsistent use of reference standards. On the other hand, the use of urine testing for cfDNA might be an alternative diagnostic tool for PTB, but our study showed that the diagnostic efficacy of this method was limited when compared to culture. A recent study showed good efficacy of cfDNA testing using urine for the diagnosis of PTB, but the study used Xpert MTB/RIF as the reference standard [39], which might lead to inconsistent results. However, the studies examined in our meta-analysis focused on adults and lacked data on children. Furthermore, the number of studies that examined cfDNA using plasma and urine for the diagnosis of PTB was limited, as was the number of included patients, so our conclusions should be treated with caution. Future large-scale multicenter studies are needed to confirm the diagnostic efficacy of cfDNA testing.

For EPTB, the categories are even richer. The studies included in this meta-analysis reported three common types of EPTB: tuberculous pleurisy, tuberculous meningitis, and abdominal TB. Our results showed that cfDNA testing had the highest diagnostic value for tuberculous pleurisy, followed by tuberculous meningitis and abdominal TB. All three types are paucibacillary and require invasive procedures to obtain specimens. Their diagnosis is difficult because of the low MTB content in the specimens. Diagnostic efficacy of nucleic acid amplification testing, represented by Xpert MTB/RIF, has remained low in most current studies of paucibacillary tuberculosis. cfDNA may be another effective method to diagnose paucibacillary tuberculosis, although current evidence remains limited. The studies included in our meta-analysis used body fluids (pleural fluid, CSF, and ascites fluid) for cfDNA testing, but not plasma specimens. We further analyzed the diagnostic efficacy of cfDNA testing in EPTB using different types of specimens. Diagnostic efficacy was better with testing plasma samples compared with non-plasma samples. However, the number of included studies was small and the results should be treated cautiously. Studies that used plasma specimens for cfDNA testing did not specifically report efficacy according to type of EPTB, so the efficacy of using plasma for cfDNA testing for different types of EPTB remains unclear. Plasma specimens are easily available and more suitable than body fluid specimens that require invasive procedures to obtain. Therefore, cfDNA plasma testing shows promise as a tool for diagnosing EPTB early. However, further studies are needed.

For different target genes, the diagnostic efficacy of IS6110 might be superior to that of other non-IS6110 target genes (such as IS1081, devR). However, the number of other non-IS6110 target genes included in this study was limited and this result needed to be treated with caution. Only one study included a subset of HIV-positive patients, but this study did not specifically report the effectiveness of cfDNA in diagnosing TB in HIV-positive patients [31]. The diagnostic efficacy of cfDNA in HIV-positive patients is still needs to be further investigated. No studies reported correlation between radiography image of patients and cfDNA test. There are no clear studies on the correlation between imaging results and cfDNA, and further studies can be conducted in this area in the future. Only a few studies had reported the concentration of cfDNA [15, 30], but these studies did not report the effect of different concentrations of cfDNA on the results of the test. Some studies had increased the concentration of cfDNA by increasing the sample volume [30], and the sample volume might be important for cfDNA concentration. However, these are still unclear and need to be confirmed by follow-up studies.

We explored the potential sources of heterogeneity in the studies in our meta-analysis using pre-identified parameters. Meta-regression analysis showed that TB type (PTB or EPTB), specimen type (plasma or non-plasma), PCR method (real-time PCR or digital PCR), and type of study design (prospective or case-control) had no effect on sensitivity and specificity. Target gene (IS6110 or non- IS6110 [IS1081, devR]) and specimen condition (fresh or frozen) affected specificity but not sensitivity. Patient selection method (convenience or consecutive) had an effect on sensitivity but not specificity. Although subgroup analysis showed significant heterogeneity in sensitivity between studies within each subgroup, sensitivity analysis did not identify specific articles as sources of heterogeneity. However, since the heterogeneity in sensitivity was significant, the results must be treated with caution. The included studies also used different CRS references and some did not include radiographic manifestations or treatment response in their CRS criteria. This might be a source of the observed heterogeneity.

There were several limitations in our meta-analysis. First, it was based on individual independent data. Some studies may be missing, despite our best efforts. Second, some studies did not report specific TB and specimen types. Third, the number of studies was limited for some subgroup analyses.

## Conclusions

This meta-analysis showed that the performance of cfDNA testing for TB diagnosis (including PTB and EPTB) was good compared with CRS and culture. There was significant heterogeneity in sensitivity and specificity. Diagnostic ability was good for both PTB and EPTB, and the overall diagnostic efficacy for each was similar. For EPTB, subgroup analysis showed that cfDNA had the highest diagnostic value for tuberculous pleurisy, followed by tuberculous meningitis and abdominal TB. Testing efficacy in EPTB was higher with plasma samples compared with non-plasma samples. cfDNA can be used for rapid early diagnosis of TB.

## Supporting information

S1 Checklist PRISMA checklist. (DOC)

S1 File Search strategy. (DOCX)

S2 File Methodological quality summary. (PDF)
