# Supplementary material for: Diagnostic accuracy of Mycobacterium tuberculosis cell-free DNA for tuberculosis: A systematic review and meta-analysis
Source: PLoS One. 2021 Jun 23;16(6):e0253658. doi: 10.1371/journal.pone.0253658 (PMC8221493; doi:10.1371/journal.pone.0253658)
Supplement: S1 File — (DOCX) [file pone.0253658.s002.docx]

Pubmed and Cochrane; The search fields were all fields.

#1 "Tuberculosis"[Mesh] OR “Tuberculoses Kochs Disease” OR “Koch's Disease” OR “Koch Disease” OR “Mycobacterium tuberculosis Infection” OR “Infection, Mycobacterium tuberculosis” OR “Infections, Mycobacterium tuberculosis” OR “Mycobacterium tuberculosis Infections”

#2 "Cell-Free Nucleic Acids"[Mesh] OR “Cell Free Nucleic Acids” OR “Nucleic Acids, Cell-Free” OR “Circulating Cell-Free Nucleic Acids” OR “Circulating Cell Free Nucleic Acids” OR “Circulating Nucleic Acids” OR “Acids, Circulating Nucleic” OR “Nucleic Acids, Circulating” OR “Cell-Free Nucleic Acid” OR “Cell Free Nucleic Acid” OR “Nucleic Acid, Cell-Free” OR “Cell-Free DNA” OR “Cell Free DNA” OR “DNA, Cell-Free” OR cfDNA OR cirDNA OR “Cell-Free Deoxyribonucleic Acid” OR “Acid, Cell-Free Deoxyribonucleic” OR “Cell Free Deoxyribonucleic Acid” OR “Deoxyribonucleic Acid, Cell-Free” OR “Circulating DNA” OR “DNA, Circulating” OR “Cell-Free RNA” OR “Cell Free RNA” OR “RNA, Cell-Free” OR cfRNA OR cirRNA OR “Cell-Free Ribonucleic Acid” OR “Acid, Cell-Free Ribonucleic” OR “Cell Free Ribonucleic Acid” OR “Ribonucleic Acid, Cell-Free” OR “Circulating RNA” OR “RNA, Circulating”

#3 #1 AND #2

Embase; The search fields were all fields.

#1 'tuberculosis'/exp OR ‘Tuberculoses Kochs Disease’ OR ‘Koch's Disease’ OR ‘Koch Disease’ OR ‘Mycobacterium tuberculosis Infection’ OR ‘Infection, Mycobacterium tuberculosis’ OR ‘Infections, Mycobacterium tuberculosis’ OR ‘Mycobacterium tuberculosis Infections’

#2 ‘Cell Free Nucleic Acids’ OR ‘Nucleic Acids, Cell-Free’ OR ‘Circulating Cell-Free Nucleic Acids’ OR ‘Circulating Cell Free Nucleic Acids’ OR ‘Circulating Nucleic Acids’ OR ‘Acids, Circulating Nucleic’ OR ‘Nucleic Acids, Circulating’ OR ‘Cell-Free Nucleic Acid’ OR ‘Cell Free Nucleic Acid’ OR ‘Nucleic Acid, Cell-Free’ OR ‘Cell-Free DNA’ OR ‘Cell Free DNA’ OR ‘DNA, Cell-Free’ OR cfDNA OR cirDNA OR ‘Cell-Free Deoxyribonucleic Acid’ OR ‘Acid, Cell-Free Deoxyribonucleic’ OR ‘Cell Free Deoxyribonucleic Acid’ OR ‘Deoxyribonucleic Acid, Cell-Free’ OR ‘Circulating DNA’ OR ‘DNA, Circulating’ OR ‘Cell-Free RNA’ OR ‘Cell Free RNA’ OR ‘RNA, Cell-Free’ OR cfRNA OR cirRNA OR ‘Cell-Free Ribonucleic Acid’ OR ‘Acid, Cell-Free Ribonucleic’ OR ‘Cell Free Ribonucleic Acid’ OR ‘Ribonucleic Acid, Cell-Free’ OR ‘Circulating RNA’ OR ‘RNA, Circulating’

#3 #1 AND #2

CNKI and Wanfang; 检索范围为“主题”

#1 结核 OR TB

#2 循环DNA OR 游离DNA OR 无细胞DNA OR cell free DNA

#3 #1 AND #2
