# Supplementary material for: Diagnostic accuracy of Mycobacterium tuberculosis cell-free DNA for tuberculosis: A systematic review and meta-analysis
Source: PLoS One. 2021 Jun 23;16(6):e0253658. doi: 10.1371/journal.pone.0253658 (PMC8221493; doi:10.1371/journal.pone.0253658)
Supplement: S2 File — (PDF) [file pone.0253658.s003.pdf]

	Risk of Bias				Applicability Concerns		
	Patient Selection	Index Test	Reference Standard	Flow and Timing	Patient Selection	Index Test	Reference Standard
Che, N. 2017	+	+	+	+	+	+	+
Han, B.a 2020	−	+	−	+	−	+	−
Han, B.b 2020	−	+	−	+	−	+	−
Li, X. 2020	+	+	+	+	+	+	+
Lyu, L.a 2020	−	+	+	+	−	+	+
Lyu, L.b 2020	−	+	+	+	−	+	+
Shao, L. 2020	+	+	+	+	+	+	+
Sharma, P. 2020	+	+	+	+	+	+	+
Shou, J. 2018	?	+	+	+	?	+	+
Yang, J.a 2017	−	+	+	+	−	+	+
Yang, J.b 2017	−	+	+	+	−	+	+
Yang, J.c 2017	−	+	+	+	−	+	+
Yang, J.d 2017	−	+	+	+	−	+	+
Yang, X. 2020	+	+	+	+	+	+	+

− **High** ? **Unclear** + **Low**

	Risk of Bias				Applicability Concerns		
	Patient Selection	Index Test	Reference Standard	Flow and Timing	Patient Selection	Index Test	Reference Standard
Labugger, I. 2017	+	+	+	−	+	+	+
Patel, K. 2018	−	+	+	+	−	+	+
Ushio, R.a 2016	−	+	+	+	−	+	+
Ushio, R.b 2016	−	+	+	+	−	+	+

− High ? Unclear + Low
